# Next generation sequencing with copy number variant detection expands the phenotypic spectrum of HSD17B4-deficiency

**DOI:** 10.1186/1471-2350-15-30

**Published:** 2014-03-06

**Authors:** Daniel S Lieber, Steven G Hershman, Nancy G Slate, Sarah E Calvo, Katherine B Sims, Jeremy D Schmahmann, Vamsi K Mootha

**Affiliations:** 1 Howard Hughes Medical Institute and Department of Molecular Biology, Massachusetts General Hospital, Boston, MA 02114, USA; 2 Center for Human Genetic Research, Massachusetts General Hospital, Boston, MA 02114, USA; 3 Department of Systems Biology, Harvard Medical School, Boston, MA 02115, USA; 4 Broad Institute of Harvard and MIT, Cambridge, MA 02141, USA; 5 Department of Neurology, Massachusetts General Hospital & Harvard Medical School, Boston, MA 02114, USA; 6 Ataxia Unit, Cognitive Behavioral Neurology Unit, Laboratory for Neuroanatomy and Cerebellar Neurobiology, Department of Neurology, Massachusetts General Hospital & Harvard Medical School, Boston, MA 02114, USA; 7 Department of Medicine, Massachusetts General Hospital, Boston, MA 02114, USA

**Keywords:** HSD17B4, DBP, D-bifunctional protein deficiency, Perrault syndrome, Next-generation sequencing, Exome sequencing, Copy number variants, CNV, Mitochondria, Mitochondrial disorders, Mitochondrial disease, Mendelian disorders, Human genetics, Ataxia, Multi-system disorders, Peroxisomal defects

## Abstract

**Background:**

D-bifunctional protein deficiency, caused by recessive mutations in *HSD17B4*, is a severe, infantile-onset disorder of peroxisomal fatty acid oxidation. Few affected patients survive past two years of age. Compound heterozygous mutations in *HSD17B4* have also been reported in two sisters diagnosed with Perrault syndrome (MIM # 233400), who presented in adolescence with ovarian dysgenesis, hearing loss, and ataxia.

**Case presentation:**

An adult male presented with cerebellar ataxia, peripheral neuropathy, hearing loss, and azoospermia. The clinical presentation, in combination with biochemical findings in serum, urine, and muscle biopsy, suggested a mitochondrial disorder. Commercial genetic testing of 18 ataxia and mitochondrial disease genes was negative. Targeted exome sequencing followed by analysis of single nucleotide variants and small insertions/deletions failed to reveal a genetic basis of disease. Application of a computational algorithm to infer copy number variants (CNVs) from exome data revealed a heterozygous 12 kb deletion of exons 10–13 of *HSD17B4* that was compounded with a rare missense variant (p.A196V) at a highly conserved residue. Retrospective review of patient records revealed mildly elevated ratios of pristanic:phytanic acid and arachidonic:docosahexaenoic acid, consistent with dysfunctional peroxisomal fatty acid oxidation.

**Conclusion:**

Our case expands the phenotypic spectrum of HSD17B4-deficiency, representing the first male case reported with infertility. Furthermore, it points to crosstalk between mitochondria and peroxisomes in HSD17B4-deficiency and Perrault syndrome.

## Background

Patients with mitochondrial disorders can present with cerebellar ataxia, often in combination with other neurological and non-neurological symptoms [1]. Such disorders can be caused by mutations in the mitochondrial DNA or by recessive, dominant, or X-linked mutations in nuclear-encoded genes essential to mitochondrial respiratory chain function [2]. Due to genetic and phenotypic heterogeneity, single gene testing is often ineffective and targeted exome sequencing is emerging as an efficient alternative [3]. Here, we report the application of such technology to a perplexing case of progressive, cerebellar ataxia in whom traditional single gene testing did not yield a molecular diagnosis.

## Case presentation

A 35-year-old man presented for evaluation of a gait disorder progressing since childhood, cognitive impairment, and sensorineural hearing loss. He had mildly delayed developmental milestones. Progressive ataxia necessitated a cane by age 18, wheelchair-dependence by 29. He needed assistance academically through school, and graduated from college at age 26. Sensorineural hearing loss was noted at age 34. Review of medical charts revealed documented azoospermia. He was the product of neurologically healthy, non-consanguineous parents, with one healthy, fertile sister.

Neurological examination revealed high arched feet, hammer toes, and normal secondary sexual characteristics. Oculomotor examination showed square wave jerks at rest, gaze-evoked nystagmus, saccadic intrusions into pursuit, hypermetric saccades, and failure to suppress the vestibular ocular reflex. There was mild dysarthria, moderate upper and lower extremity dysmetria, and moderate gait ataxia (Brief Ataxia Rating Scale: 13/30). Deep tendon reflexes were normal in the arms, exaggerated in the legs. Plantar responses were flexor. He had impaired pin sense in the feet. Hearing was intact on office testing. He had an average IQ (FSIQ = 98, 45th percentile, WAIS-III), bland affect, reduced visual motor processing speed, and was impaired on a card-sorting task, a nonverbal test of concept formation and cognitive flexibility, with perseverative errors and trouble maintaining set.

**Figure 1:**
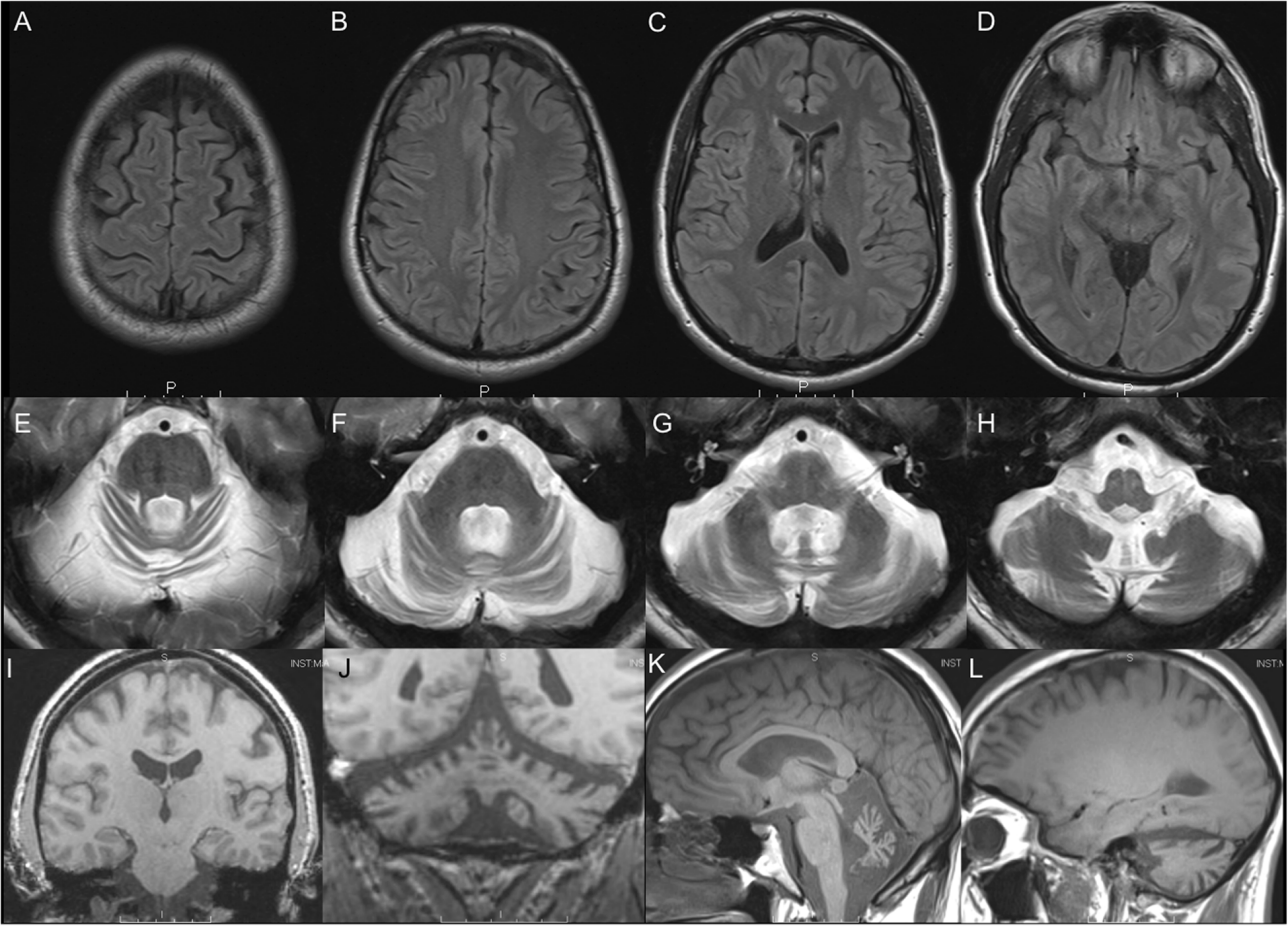
**MRI at age 35 demonstrating cerebellar volume loss with preservation of cerebral hemispheres. A-D)** FLAIR axial; **E-H)** T2 axial; **I-J)** Coronal T1; **K-L)** Sagittal T1.

Brain MRI from age 14 through 35 showed progressive cerebellar volume loss (Figure 1). Nerve conduction studies revealed absent long latency responses in the legs, symmetrically reduced motor conduction velocities in arms and legs with normal amplitudes; needle examination was normal. Waking electroencephalogram was normal. Audiology testing revealed mild decrease in hearing for higher frequencies bilaterally, decreased speech intelligibility, and word recognition 86% on the right, and 94% on the left.

Laboratory data included normal routine tests and negative genetic tests for 18 mitochondrial or ataxia genes (See Additional file 1). Testosterone was low at 164 ng/dL (270–1100) and follicle-stimulating hormone (FSH) was elevated at 15 mIU/mL (1–8). Plasma lactate was within normal limits, while pyruvate was essentially normal at 0.18 mmol/L (0.08-0.16). Urine organic acid analysis showed small amounts of lactate in urine. Tests of long and very long chain fatty acids revealed elevated total plasma ω-9 fatty acids (500 ug/mL or 24.07% of total fatty acids; reference range: 15.4%-22.0%). The percentage of C18:2ω6 (linoleic) was low (490 ug/mL or 23.6% of total; range: 24.2%-33.8%), and the ratio of pristanic acid/phytanic acid (0.19; range: 0.01-0.18), percentage of arachidonic acid (208 ug/mL or 10.0% of total; range: 4.0%-9.5%), and ratio of arachidonic/docosaehexenoic acid (5.6; range: 0.43-5.4) were mildly elevated.

Muscle biopsy appeared unremarkable on light and electron microscopy. Muscle coenzyme Q10, free and total carnitine, and acylcarnitine were normal, but electron transport chain testing of a skeletal muscle biopsy (Additional file 1) revealed mild Complex I deficiency (22% Complex I + III after normalization to citrate synthase).

## Results

Given the clinical suspicion of mitochondrial disease, we performed targeted exome sequencing as previously described, interrogating the entire mitochondrial DNA (mtDNA) and the exons of 1598 nuclear genes implicated in mitochondrial disease, mitochondrial function, or other disorders with phenotypic overlap [3]. We identified 1,569 single nucleotide variants (SNVs) and small insertions/deletions (indels) in the patient’s sample. We searched for pathogenic mtDNA variants as well as autosomal recessive, dominant-acting, or hemizygous X-linked variants but did not identify a likely genetic cause of disease [3].

**Figure 2:**
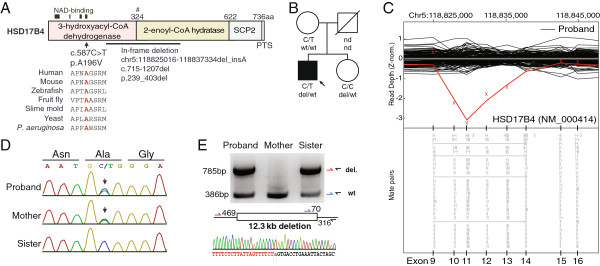
**Compound heterozygous variants in *HSD17B4*. A)** Schematic of HSD17B4 (NP_000405) with multiple sequence alignment. SCP2, sterol carrier protein 2; #, cleavage site. **B)** Pedigree denoting genotypes. **C)** Normalized depth of coverage at each exon (“x”); lines represent smoothed data from proband (red) and controls (black). Below, reads showing deletion-spanning mate pairs. **D)** Sanger trace of c.587C > T. **E)** DNA gel indicating genotypes at deletion site, schematic of primers, and Sanger trace across deletion in proband. Abbreviations: wt, wildtype; nd, no data; del, deletion; norm, normalized.

To complement the SNV and indel analysis, we applied CONIFER to identify large copy number variants (CNVs) in our targeted exome data [4]. CNV analysis revealed a potential 13 kb heterozygous in-frame deletion of exons 10–13 of *HSD17B4* (c.715-1207del; p.239_403del) (Figure 2)*.* Review of patient exome data revealed that the patient also harbored a rare, heterozygous missense variant in *HSD17B4* (c.587C > T; p.A196V). The SNV was not present in Exome Variant Server nor 1000 Genomes, altered a residue conserved to bacteria within a predicted NAD-binding domain, and is predicted to be “probably damaging” by PolyPhen2. Familial genotyping confirmed the deletion was inherited from the father, and compounded with a missense mutation inherited from the mother (Figure 2).

Recessive mutations in *HSD17B4* have previously been associated with infantile D-bifunctional protein (DBP) deficiency (OMIM #261515) a severe disorder of peroxisomal fatty acid beta-oxidation that is generally fatal within the first 2 years of life [5]. Recessive *HSD17B4* mutations were more recently identified as the cause of Perrault syndrome (OMIM #233400) in two sisters who fulfilled the defining features of the syndrome (ovarian dysgenesis and sensorineural deafness) and who also presented with peripheral neuropathy and ataxia [6]. Due to the phenotypic overlap with the latter cases, the predicted severity of the *HSD17B4* mutations, and biochemical evidence of altered peroxisomal fatty acid metabolism, we reached a diagnosis of HSD17B4 deficiency in our patient.

## Discussion and conclusions

HSD17B4, also known as D-bifunctional protein (DBP), is a peroxisomal enzyme that catalyzes multiple steps of beta-oxidation of very long chain fatty acids (VLCFA). While named for its homology to a steroid-converting enzyme, the protein’s role in steroid hormone metabolism is unclear [7]. The protein consists of three domains: an N-terminal dehydrogenase domain, a hydratase domain, and a sterol carrier protein (SCP) domain. The three amino acids at the C-terminus (“AKL”) constitute the peroxisomal targeting signal (PTS). After peroxisomal import, the 79-kDa full-length protein is proteolytically cleaved to yield a 35-kDa dehydrogenase subunit and a 45-kDa hydratase subunit containing the hydratase and SCP domains [8].

Recessive mutations in HSD17B4 are known to cause DBP-deficiency, an early-onset neurological disorder characterized by neonatal hypotonia, seizures, visual impairment, and psychomotor retardation [5]. Patients with the disorder generally die in infancy, although a small number survive into their teens [9]. Like our patient, individuals with DBP deficiency often show an elevated ratio of pristanic to phytanic acid in plasma, reflective of dysfunctional beta-oxidation in the peroxisome [10].

DBP-deficiency has been classified into subtypes based on the affected enzymatic activities of HSD17B4. Type I patients have deficiencies of both the hydratase and dehydrogenase subunits whereas types II and III have isolated hydratase or dehydrogenase deficiency, respectively. Recently, a Type IV category was proposed to describe mildly affected patients with compound heterozygous mutations affecting both subunits [11]. Our case may be classified as type IV due to the mutations affecting both subunits and the phenotypic similarity between our patient and two teenage brothers categorized as type IV, including the presence of cerebellar ataxia, polyneuropathy, pes cavus, and hearing loss [11].

Recently, recessive mutations in *HSD17B4* have been identified in two sisters as a cause of Perrault syndrome (OMIM #233400). Perrault syndrome was first defined in 1951 as ovarian dysgenesis and sensorineural hearing loss in females [12]. Brothers of Perrault syndrome females have been reported with hearing loss but are reportedly fertile [13]. Perrault syndrome is genetically heterogeneous and recessive mutations in three additional genes have been implicated to date, including *HARS2*, *LARS2*, and *CLPP*, which respectively encode the mitochondrial histidyl- and leucyl-tRNA synthetases and a mitochondrial ATP-dependent protease [14-17].

The genotype and phenotype of our patient resemble that of the two sisters recently diagnosed with Perrault syndrome [6]. All three cases harbored compound heterozygous *HSD17B4* mutations affecting the dehydrogenase and hydratase subunits. Both our patient and the older of the two sisters presented with ataxia, demyelinating polyneuropathy, pes cavus, and hearing loss, with marked cerebellar atrophy on MRI. Although levels of VLCFA levels were reportedly normal in the older sister [18], our patient had alterations compatible with DBP deficiency. While the previously reported sisters [6] presented with ovarian dysgenesis, an essential characteristic of Perrault syndrome, our male patient has azoospermia with normal secondary sexual characteristics. Our case raises the hypothesis that azoospermia can be a feature of HSD17B4 deficiency in males, consistent with the finding that *HSD17B4* knockout mice exhibit male-specific sterility with testicular lipid accumulation [19].

The mechanisms linking HSD17B4 deficiency and azoospermia remain unclear. Our patient had low testosterone levels, combined with an elevated FSH, suggesting primary testicular failure. HSD17B4 was initially thought to be a steroid-converting enzyme due to its sequence homology, and the enzyme has been shown to display multifunctional properties, including both fatty acid and estradiol oxidation [7,20]. However, in vivo studies and computational evidence have suggested that its primary role is likely in fatty-acid metabolism, while steroid conversion is only a secondary and possibly minor activity in vivo [21]. Evidence in mice suggests that HSD17B4 may play an important role in lipid homeostasis in the testes, specifically in Sertoli cells [19]. Further investigation is required to determine how HSD17B4 deficiency may lead to male infertility and low testosterone in humans.

The current case underscores clinical and biochemical overlap between mitochondrial and peroxisomal disorders. Our patient’s clinical presentation raised suspicion of mitochondrial disease, leading to a skeletal muscle biopsy that demonstrated mild respiratory chain deficiency. Yet the genetic lesion lies within HSD17B4, a protein classically annotated as being peroxisomal based on detailed localization studies [7]. Interestingly, HSD17B4 is also found within MitoCarta, a large-scale inventory of the mitochondrial proteome [22], suggesting that the protein may be dual localized. The other three genes associated with Perrault syndrome (*HARS2*, *LARS2*, *CLPP*) are also present in MitoCarta, were targeted for sequencing, and no rare, protein-modifying variants were present in the patient. Future studies will be required to determine whether HSD17B4 can indeed be dual-localized to mitochondria and peroxisomes. If so, HSD17B4 would be added to a small but growing list of disease gene products that can be dual localized to these two compartments (e.g. AGXT, MPV17) [23,24]. An alternative explanation may be that defective peroxisomal fatty acid catabolism can lead to secondary mitochondrial dysfunction [25], indicating biochemical crosstalk across the two organelles.

### Consent

Study protocols were approved by the Partners Human Research Committee. Written informed consent was obtained for publication of this case report and any accompanying images.

## Abbreviations

mtDNA: Mitochondrial DNA; MRI: Magnetic resonance imaging; DBP: D-bifunctional protein; OMIM: Online Mendelian inheritance in man; CNV: Copy number variant; PTS: Peroxisomal targeting signal; VLCFA: Very-long-chain fatty acid.

## Competing interests

The authors declare that they have no competing interests.

## Authors’ contributions

JDS looked after the patient. DSL, SGH, and SEC performed sequence analysis. NGS, KBS aided in data analysis. JDS, VKM supervised data analysis and interpretation. DSL, SGH, JDS, and VKM wrote the report. All authors contributed to revising the manuscript and approved the final version.

## Authors’ information

Jeremy D Schmahmann and Vamsi K Mootha contributed equally to this work as co-senior authors.

## Pre-publication history

The pre-publication history for this paper can be accessed here:

http://www.biomedcentral.com/1471-2350/15/30/prepub

## Supplementary Material

Additional file 1 Supplementary materials including experimental methods, clinical information, and a table of biochemical assay results. Click here for file
