# Supplementary material for: Next generation sequencing with copy number variant detection expands the phenotypic spectrum of HSD17B4-deficiency
Source: BMC Med Genet. 2014 Mar 6;15:30. doi: 10.1186/1471-2350-15-30 (PMC4015298; doi:10.1186/1471-2350-15-30)
Supplement: Additional file 1 — Supplementary materials including experimental methods, clinical information, and a table of biochemical assay results. [file 1471-2350-15-30-S1.pdf]

Supplementary Materials

Lieber et al., "Next generation sequencing with copy number variant detection expands the phenotypic spectrum of HSD17B4-deficiency"

Supplementary Methods

Genotyping

PCR of deletion (hg19 chr5:118825016-118837334del_insA; NM_000414:c.715-1207del) was performed using AccuPrime kit (Life Technologies) with an extension time of 80s and annealing temperature of 55ºC. The following primers were used, as depicted in Figure 2E: GGGAATGAATGGTACCCAGAT (red), GCTTTCATTTTGAACATGGTTG (blue), TTTTACCATCCACAGGCTCAC (black).

AccuPrime PCR of SNV (NM_000414:c.587C>T) was performed using an extension time of 1 minute and an annealing temperature of 54ºC. The following primers were used: AGTTGCTTTTGATAGGTGCAG (forward) & CCCCATTGTGTA AAACAAAAA (reverse).

Multiple sequence alignment

Multiple alignment of HSD17B4 protein in Figure 2A was created by aligning the following Uniprot IDs with ClustalW[1]: P51659, P51660, Q98TA2, Q9NKW1, Q9VXJ0, Q02207, Q9I4V1.

Variant annotation

Genetic variants were annotated with predictions from PolyPhen2[2], and variant frequencies from Exome Variant Server[3] and 1000 Genomes[4].

Fatty acid testing

Fatty acid testing was performed at the Kennedy Krieger Institute in Baltimore, MD using capillary gas chromatography / mass spectroscopy of pentafluorobenzyl bromide fatty acid esters.[5] Reference range was defined as within two standard deviations of mean in adult controls.

Electron Transport Chain (ETC) Testing

ETC testing of muscle tissue was performed at the Center for Inherited Disorders of Energy Metabolism (CIDEM) at Case Western Reserve University, Cleveland, Ohio. <20% activity of any complex after normalization to citrate synthase was considered a major deficiency and 20-30% activity was considered a minor deficiency, as previously described.[6]

Supplementary Clinical Information

Developmental delay includes sitting at 8 months, walking at 14 months, talking at 18 months, and difficulty reading at age 6. Running was impaired at age 7, and sports impossible by age 9.

Pertinent normal laboratory studies included thyroid functions, vitamins E and B12, copper, ceruloplasmin, serum protein electrophoresis, serum tissue transglutaminase and gliadin antibodies, rapid plasma reagin (RPR), and antinuclear antibodies (ANA). Normal genetic tests included *SCA1, SCA2, SCA3, SCA5, SCA6, SCA7, SCA8, SCA10, SCA14, SCA17, DRPLA, FRDA, AOA1, AOA2, POLG, MELAS, MERRF* and *NARP*. Endocrine tests with normal results were luteinizing hormone (LH) 11 mIU/mL (2-12), somatomedin 112 ng/mL (114-492), and prolactin at 11 ng/mL (2-19). Elevated serum amino acids included alanine 638 umol/L (146-494), asparagine 121 umol/L (26-92), glutamic acid 75 umol/L (6-62), isoleucine 103 umol/L (39-90), lysine 260 umol/L (119-243), methionine 38 umol/L (13-37), proline 310 umol/L (97 – 297), and valine 365 umol/L (172 – 335).

Table S1. Results of electron transport chain (ETC) assays of muscle tissue.

**Assay**	**Complex or Enzyme**	**Patient**	**Mean (Controls)**	**Range (Controls)**	**Patient / Mean**
NADH-cyt. *c* reductase (rotenone sensitive)	Complex I,III	0.1	1.2	0.2-4.7	11.2%
NADH-ferricyanide reductase	Complex I	20	29.9	11.5-60.1	66.8%
Succinate-cyt. *c* reductase (antimycin sensitive)	Complex II,III	0.4	2.1	0.4-4.9	18.3%
Succinate dehydrogenase	Complex II	0.6	0.8	0.1-2.0	70.7%
Decylubiquinol-cyt. *c* reductase	Complex III	6.7	15.2	6.8-35.2	43.9%
Cytochrome *c* oxidase	Complex IV	37	148.9	57.3-373.0	24.9%
Citrate synthase	CS	10.1	18.6	9.4-30.0	54.3%

References

1. Thompson JD, Gibson TJ, Higgins DG. Multiple sequence alignment using ClustalW and ClustalX. Current protocols in bioinformatics / editoral board, Andreas D Baxevanis [et al] 2002;Chapter 2:Unit 2 3 doi: 10.1002/0471250953.bi0203s00[published Online First: Epub Date]|.

2. Adzhubei IA, Schmidt S, Peshkin L, Ramensky VE, Gerasimova A, Bork P, Kondrashov AS, Sunyaev SR. A method and server for predicting damaging missense mutations. Nature methods 2010;**7**(4):248-9 doi: 10.1038/nmeth0410-248[published Online First: Epub Date]|.

3. Exome Variant Server, NHLBI Exome Sequencing Project (ESP), Seattle, WA (URL: http://evs.gs.washington.edu/EVS/) [January 2012].

4. The 1000 Genomes Project Consortium. A map of human genome variation from population-scale sequencing. Nature;**467**(7319):1061-73 doi: nature09534 [pii] 10.1038/nature09534[published Online First: Epub Date]|.

5. Lagerstedt SA, Hinrichs DR, Batt SM, Magera MJ, Rinaldo P, McConnell JP. Quantitative determination of plasma c8-c26 total fatty acids for the biochemical diagnosis of nutritional and metabolic disorders. Molecular genetics and metabolism 2001;**73**(1):38-45 doi: 10.1006/mgme.2001.3170[published Online First: Epub Date]|.

6. Bernier FP, Boneh A, Dennett X, Chow CW, Cleary MA, Thorburn DR. Diagnostic criteria for respiratory chain disorders in adults and children. Neurology 2002;**59**(9):1406-11
